# Reproducing burrows in modelled sedimentary strata

**DOI:** 10.1038/s41598-024-52333-8

**Published:** 2024-02-06

**Authors:** Hassan Eltom

**Affiliations:** 1 Geosciences Department, College of Petroleum Engineering Geosciences, King Fahd University of Petroleum Minerals, Building 76, Dhahran, 31261 Kingdom of Saudi Arabia; 2 Center for Integrative Petroleum Research, College of Petroleum Engineering Geosciences, King Fahd University of Petroleum Minerals, Building 78, Dhahran, 31261 Kingdom of Saudi Arabia

**Keywords:** Ecology, Environmental sciences, Hydrology, Solid Earth sciences, Energy science and technology, Carbon capture and storage, Energy harvesting, Energy infrastructure, Energy storage, Fossil fuels

## Abstract

Studying bioturbated sedimentary strata is crucial; however, sampling these strata poses notable challenges. Modelling these strata has emerged as a promising solution to bridge this gap. This study introduces a workflow to model burrows utilizing the multipoint statistics (MPS) method. A key step in MPS modelling is the use of training images, and this study describes a process to create them using CT scans of rock samples contain burrows. These scans give a 3D visual representation of burrows in actual rock record. The process involves selecting suitable rock samples, CT scanning them, importing and processing the scans in Petrel™, and then transforming the scan data into training images which can be used for MPS modelling. The MPS models allow for precise replication of burrows, variations in their size and percentage, and modeling properties like porosity and permeability. This enables a more detailed analysis, paving the way for further advancements in understanding and simulating the geological implications of burrows. To guarantee reproducibility, this study has precisely documented the workflow with video guidance and provided the necessary data. This comprehensive documentation aims to encourage the broader adoption of MPS modelling for bioturbated strata, setting the stage for further advancements in the field.

## Introduction

Burrows in stratigraphic record^1–3^ can be studied for various geological applications, including paleoenvironmental analysis, interpretation of depositional environments, and assessing the quality of reservoirs and aquifers^4–8^. In ichnological studies, particularly those focusing on depositional interpretation, stratigraphic surfaces, and paleoenvironmental analysis, two-dimensional (2D) views of burrows are often sufficient and can provide valuable insights. These 2D perspectives can effectively capture essential data in numerous contexts. However, in certain specialized fields, especially in petroleum and hydrogeological research, three-dimensional (3D) views of burrows become indispensable. This is primarily because a 3D perspective is crucial for evaluating aspects like burrow connectivity and their potential to form permeable pathways^9–16^.

CT scans offer a detailed 3D evaluation of burrows, a process essential for accurately predicting their impact on fluid flow and storage in subsurface environments^17–19^. Yet, this approach is not without its challenges. Data from CT scans often face limitations due to size dependencies, and finding samples that represent burrow connectivity accurately is extremely rare. Furthermore, even when such samples are available, their large number and volume present substantial challenges for laboratory analysis. Consequently, this leads to a fragmented understanding of the petrophysical properties in reservoirs and aquifers with burrows, affecting the accuracy of their models, ultimately impacting the exploitation of their natural resources. The presence of such limitations necessitates the development of more sophisticated modeling techniques. Digital rock modeling and analysis, in this regard, offer a promising solution to navigate these complexities.

Earlier studies tried to model burrows but often showed them as simple cylinders, missing their complex shapes^9–11, 20–22^. Recently, a series of comprehensive studies has significantly advanced the modelling of burrows through the application of multipoint statistics (MPS) and various cutting-edge software packages^19,23–27^. These works have led to remarkable progress in this field. One of the notable achievements of MPS modelling is the ability to capture realistic geometrical patterns in the burrows, closely resembling those found in the actual stratigraphic record. This has been a critical, as it provides a more accurate representation of burrow structures compared to previous methods. MPS models have also introduced the use of geocellular grids (enabling the incorporation of realistic and randomized distribution of petrophysical properties within the burrow models). Unlike earlier approaches that assigned single values of porosity and permeability for the entire burrow network and uniform values for the surrounding medium, this new approach more realistically accounts for the heterogeneity present realistic models for sedimentary strata. Furthermore, these numerical models have opened up new possibilities for advanced investigations, such as conducting fluid flow simulations on the modelled burrows. Such advancement allows researchers to gain valuable insights into the fluid flow characteristics within the burrows, leading to a deeper understanding of their geological implications. The outcomes of these studies offer more accurate and comprehensive insights, paving the way for further advancements in investigating the geological significance of burrows.

**Figure 1:**
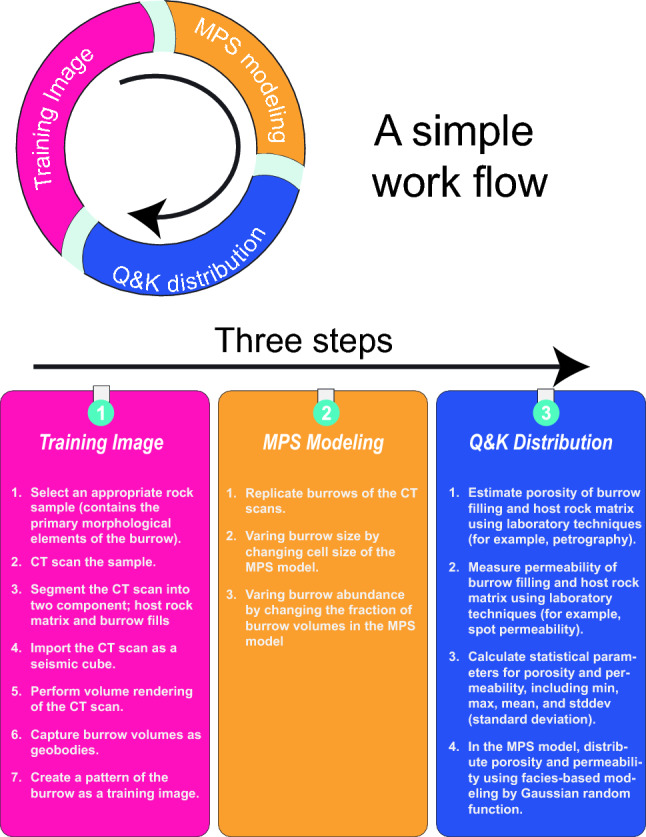
Infographics detailing the workflow steps for generating multipoint statistics models of burrows.

To promote the widespread adoption of MPS modelling in ichnological studies (specially studying burrow-related strata), this paper presents a comprehensive step-by-step workflow that instructs researchers on generating MPS models of burrows (Fig. 1). The provided instructions are complemented by accompanying videos illustrating the execution of each step, ensuring clarity and ease of implementation. Additionally, the study includes the necessary supplementary data to facilitate reproducibility. The outlined workflow is not only highly reproducible but also user-friendly, even though it comprises multiple steps. Its accessibility enables researchers to research into the promising realms of ichnology and exploration geology, as MPS modelling helps overcome the challenges associated with sampling burrowed sedimentary strata. Offering such a novel approach to understanding and simulating burrows paves the way for exciting advancements in the ichnology field.

## Training image

**Figure 2:**
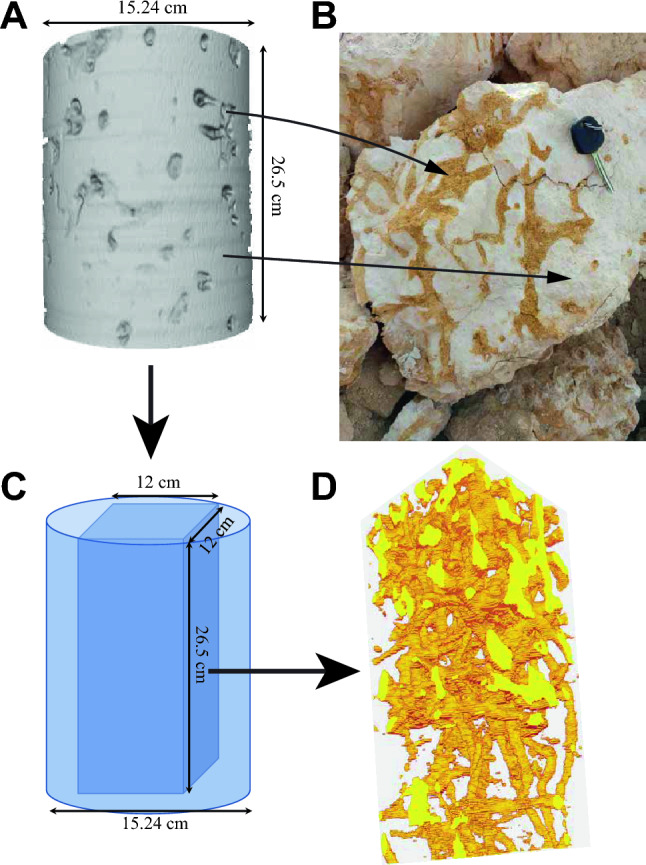
Screen captures and a photograph illustrating the process for the generation of training images and multipoint statistics modelling of burrows from CT scan. Panel (**A** and **B**) highlights the pronounced image contrast between the burrow fill and the surrounding host rock matrix, attributed to their density differences, which enhances CT scan resolution and facilitates capturing of the burrow network (**C** and **D**). The CT scan data is for a core extracted from the Hanifa Formation in central Saudi Arabia.

To create effective MPS models for burrows that replicate patterns in the stratigraphic record, it is crucial to base them on realistic training images. Much of the MPS modelling effort centers on ensuring the reality of these images. In this context, the author presents a workflow for crafting training images of burrows using CT scans (Fig. 2). The training image produced from CT scans (Fig. 2A) offers a 3D visual representation of burrow patterns (Fig. 2D). This approach provides a more authentic representation compared to conceptual drawings and 2D tracings of photographs due to its ability to capture the 3D patterns of burrows from actual rock structures. However, creating this training image involves a complex process that demands careful adherence (Fig. 1). The specific steps for generating this pattern are outlined in the following section and documented in Videos S1–S5.

### Rock samples

First, researchers should carefully select rock samples containing burrows that exhibit clear distinctions from the surrounding host rock matrix (Fig. 2). For example, burrows such that in a *Glossifungites* ichnofacies with passive filling in mud-dominated host rock matrix^14^ and those with contrasting minerology such as dolomite burrow fills and calcite host rock matrix^11,12^ often display unique burrow morphologies (Fig. 2). The sample size of such burrows must be sufficiently large to encapsulate the key morphological features of the burrows. For instance, in the case of *Thalassinoides*, it is crucial that the sample includes burrow volume that represent the boxwork pattern of the burrow such as angular connections and shafts and tunnel interconnections. It is not necessary, however, for the sample to demonstrate the complete connectivity of the burrows.

The distinct features of burrows can be identified in the CT scans of the rock samples, primarily based on the density contrast between the burrow filling and the host rock matrix (Fig. 2). Such CT scans (example in supplementary files S1) serve as crucial input to generate the training image necessary for the accurate and effective burrow modelling process (Fig. 2). Although the workflow introduced in this paper uses an example of a single burrow, it is important to note that the methodology can be extended to model multiple burrows. The adaptability of the presented workflow allows researchers to apply it effectively for the simulation of multiple burrows.

### CT scanning

Upon collecting the rock sample containing burrows (Fig. 2), this sample should undergo CT scanning using medical or micro CT scanning. Many published works have described how rock CT scanning of burrow-related strata is performed^11,13, 17, 28^. For more details about rock CT scanning, the readers are referred to these examples. An important note about CT scanning of the sample containing burrows is that it should be performed with a resolution that can capture the burrow network in the rock (Fig. 2D).

### Importing CT scans volume in petrel

**Figure 3:**
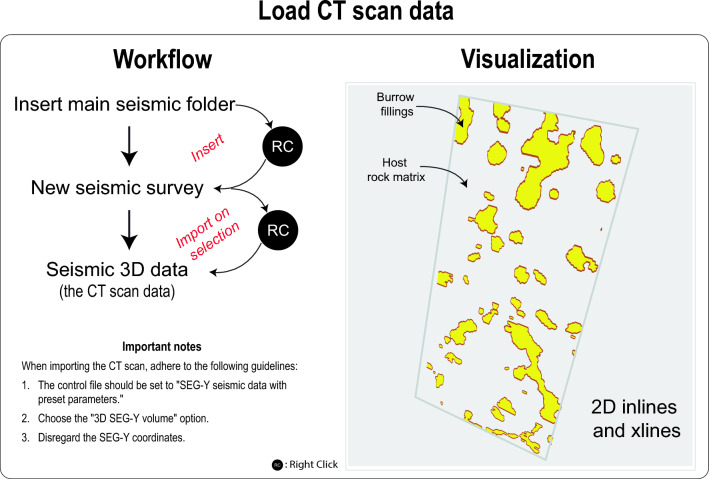
Workflow and visualization of loading the CT scan data in Petrel.

**Figure 4:**
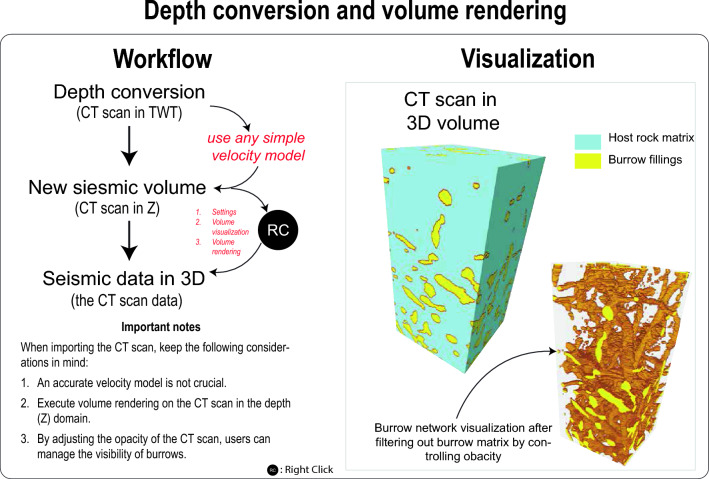
Workflow and visualization of depth conversion and volume rendering.

The CT scans of the rock sample need to be segmented into two distinct rock textures: 1) the burrows and 2) the host rock matrix (Figs. 2, 3). This segmentation process can be carried out prior to importing the CT scan into Petrel, using software such as PerGeos or 3DSlicer. Once the CT scans of the rock have been successfully segmented, they can be imported into Petrel as a seismic cube, utilizing the SEQ-Y file format (Fig. 3, Video S1). The CT scan data, functioning as a seismic cube, is initially presented in time domain. To facilitate the processing of these CT scans for the creation of a training image in the ensuing stages, a transformation is necessary. This involves converting the CT scan data from the time domain to the depth domain, achieved through the utilization of a velocity model (Fig. 4, Video S2). The conversion can be efficiently executed within Petrel, utilizing the domain conversion module (Fig. 4, Video S2). In this context, it is important to note that precision is not a paramount concern for this conversion process. Users have the flexibility to adopt a straightforward velocity model, as illustrated in Video S2, to accomplish this step.

### Volume rendering

After completing the domain conversion (Fig. 4) process of the CT scan data, the user can proceed to the next step of volume rendering (as demonstrated in Video S3). Volume rendering is a crucial technique for visualizing the 3D dataset obtained from the CT scans. This technique involves the creation of a 2D image that effectively represents the intricate 3D structures within the dataset. Unlike traditional 2D visualization methods, volume rendering offers the advantage of showcasing complex geological structures and phenomena such as burrow network with greater clarity (Fig. 4). The user can initiate the volume rendering process by referring to the procedures shown in Video S3. Upon successful execution, the output will be a comprehensive 3D representation of the burrow network (Fig. 4) within the CT scan data.

### Capturing burrows as geobodies

**Figure 5:**
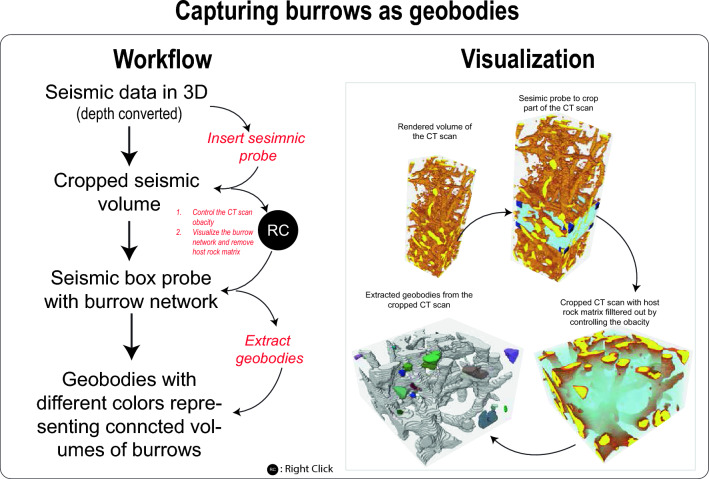
Workflow and visualization of capturing burrows as geobodies.

In order to accurately capture the complex morphology of the burrows, it is recommended to utilize a small-sized CT scan volume (Fig. 5). In cases where a larger CT scan is employed (as illustrated in the demonstration Video S4), the scan should be cropped accordingly (Fig. 5). To achieve this, a seismic box probe can be applied to isolate a cropped CT scan volume that encompasses a simplified representation of burrows, as clearly depicted in Fig. 5 and Video S4. By manipulating the opacity settings of the CT scan within the seismic box probe, it becomes possible to selectively filter out the host rock matrix, allowing for an isolated visualization of the burrows on the screen (Fig. 5, Video S4). Subsequently, this refined volume of burrows can be extracted as geobodies, a process detailed in Video S4. Among these extracted geobodies, the largest one typically spans the entirety of the box probe, serving as a fundamental component in the subsequent construction of the training image.

**Figure 6:**
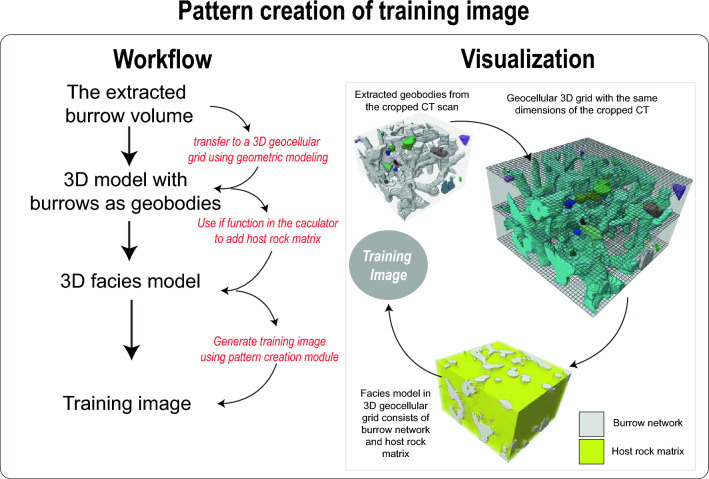
Workflow and visualization of pattern creation of training image.

In this phase, the user proceeds to transfer the extracted largest volume of burrows (referred to as the volume of burrows) onto a geocellular grid (Fig. 6), as clearly demonstrated in Video S4. A geocellular grid as defined here in this study is a 3D model composed of discretized cells, used in geology and the oil and gas industry to represent and analyze subsurface structures and properties. It is important to ensure that the geocellular grid possesses identical volume and dimensions to the seismic box probe from which the geobody was extracted (Fig. 6). Moreover, the cell volume of the geocellular grid should be closely aligned with the dimensions of the individual voxels within the geobody. This careful alignment facilitates the creation of a geobody within the geocellular grid that remains consistent with the characteristics of the burrow CT scans (Fig. 6), as explicitly depicted in Video S4.

### Pattern creation of training image

The user has the option to transform the extracted geobody into representations of burrows by assigning it a specific facies code as shown in Fig. 6 and Video S5. Likewise, the encircling cells bordering the burrows can be designated with a distinct code to denote the host rock matrix (Video S5). This strategic allocation of facies codes yields the creation of a geocellular grid characterized by two clearly differentiated rock textures (Fig. 6, Video S5). Such a configuration is the requisite input for generating patterns that form the basis of a training image through the employment of the pattern creation module in Petrel, a process comprehensively illustrated in Video S5. The generated training image can undergo testing, allowing for a comprehensive assessment. The outcomes derived from this testing phase can then be visually compared to the original CT scan volume, enabling a thorough qualitative evaluation (Video S5). The training image appears as a multipoint facies pattern positioned beneath the extracted geobody within Petrel’s interface.

## MPS modelling

**Figure 7:**
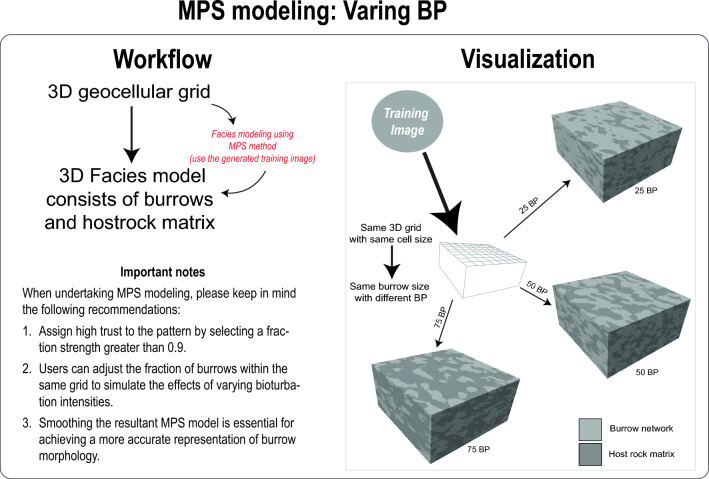
Workflow and visualization of varying burrow percentage (BP) using MPS modeling.

**Figure 8:**
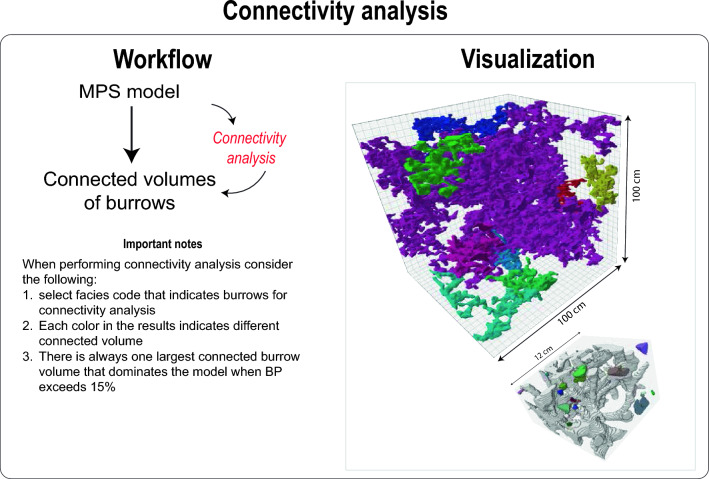
Workflow and visualization of connectivity analysis of the results from MPS modelling.

**Figure 9:**
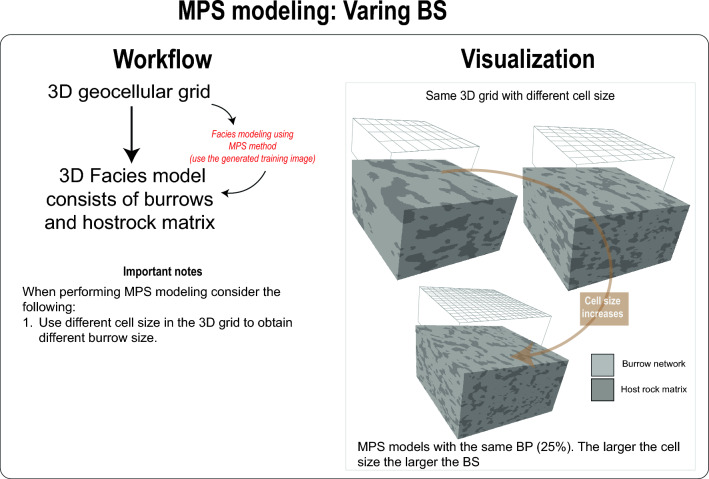
Workflow and visualization of MPS modelling when variation burrow percentage (BP).

Utilizing the training image, burrows from CT scans can be precisely replicated using MPS modelling (Figs. 7, 8, 9, Videos S6). Not only does this method allow for adjustments in burrow size and percentage (as shown in Videos S7, S8, and Figs. 7, 8), but it also facilitates the creation of digital rock samples showcasing varied burrow characteristics—something that is tough to obtain from real samples. Further, these MPS models enable the generation of petrophysical models, capturing the heterogeneity observed in actual rock samples (Video S9). Such models can be used in fluid flow simulation in advanced steps.

### Replicating burrows of the CT scans

The generated training image can serve as input for precise replication using MPS modelling, as demonstrated in Video S6. For this task, a 3D grid of dimensions 1000 × 1000 × 1000 units (whether m, cm, mm, or µm) can be established. The cell size within this grid can be set at 2 unit^3^, as shown in Video S6, leading to a grid comprising 125 × 10^6^ cells. By using Petrel’s facies modelling and selecting MPS modelling, users can reproduce burrows within this 3D grid (Figs. 7, 8, 9). Once MPS modelling concludes, a connectivity analysis can be executed to observe the varying connected volumes of burrows (Fig. 8). This often results in identifying one predominant connected burrow volume and several significantly smaller unconnected burrow volumes. Upon visualizing these volumes (Video S6), similarities in pattern and morphology between the burrows in the CT scans and the MPS models become evident (Fig. 8). This underscores the effectiveness of the approach presented in this study to accurately replicate burrows as seen in the rock record (Fig. 8).

### Varying burrow percentage in MPS models

Within a single 3D grid (Fig. 7), the user has the flexibility to adjust the burrow percentage (Video S8). In MPS modelling, the default percentage of burrows corresponds to that of the training image. This percentage can be manually modified by the user (Fig. 7), and Petrel aims to reproduce burrows closely matching the specified proportion. A warning message will appear if the specified percentage deviates beyond a 10 percent tolerance, but this won’t halt the modelling process.

### Varying burrow size MPS models

The cell size within the 1000 × 1000 × 1000 grid can be adjusted (Fig. 9). For instance, as detailed in the above sections, a cell size of 2 unit^3^ resulted in a total of 125 × 10^6^ cells. By increasing the cell size to 5 unit^3^, the total cell count dramatically decreases to 8 × 10^6^ cells (Fig. 9). When MPS modelling is executed on a 3D grid with a cell size of 5 unit^3^, the resultant burrows are significantly larger than those in the model with a cell size of 2 unit^3^. See video S7 for the demonstration of this practice. Thus, the user can control burrow size in the MPS model by adjusting the cell size and fix the volume of the model (Fig. 9).

### Distributing porosity and permeability in the MPS models

**Figure 10:**
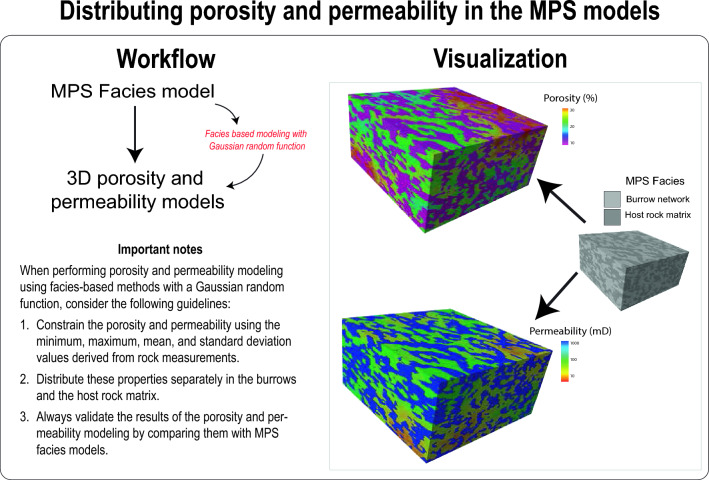
Workflow and visualization of distributing porosity and permeability in MPS models.

Since MPS models are built on geocellular grids, distributing petrophysical properties across these grid cells can be done using different methods, yet within realistic statistical constraints (Video S9). In Petrel, users can use the Gaussian random function simulation to distribute petrophysical properties randomly across the MPS model cells (Fig. 10). This distribution is bound by specified ranges, means, and standard deviations of properties. Users should initially determine these parameters in the lab or derive them from analogues (Fig. 10). This approach yields geologically consistent distributions for both the modelled burrows and their surrounding host rock matrix, as illustrated in Video S9.

## Discussion

### Numerical modelling of burrows

**Figure 11:**
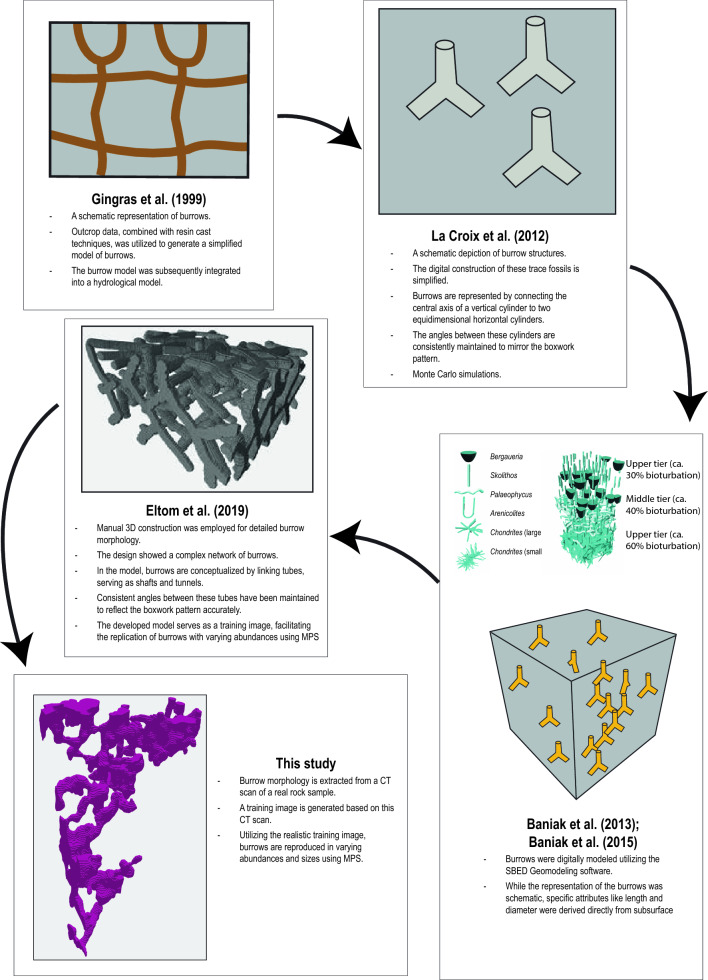
An illustration comparing the morphological modelling of burrows in previous works with the approach used in this study.

The field of numerical modelling of burrows remains relatively underexplored^12^. There are key studies^10,11, 19, 21, 22, 26, 27, 29^ that have concentrated on the modelling of burrow structures and their implications for petrophysical properties (Fig. 11). Yet, given the significance of burrows in controlling petrophysical properties of sedimentary strata there is a pronounced need for an expanded and rigorous research in this area.

The typical approach to modelling burrowed strata involves three main steps. First, constructing a 3D grid to represent the host rock matrix with specific reservoir properties. Second, populating this grid with burrows of varied volumes and dimensions, each possessing distinct reservoir properties, such as porosity and permeability, relative to the host matrix. Third, conducting fluid flow simulations within this framework. Although many of previous studies adopt this foundational framework, their methodologies diverge notably in the detailed representation of burrow morphology.

The difference in methodologies for burrow morphology representation significantly affects the reliability and interpretability of the simulation outputs for modelled burrowed strata. In the subsequent discussion, the author systematically reviews prior studies, examining their specific modelling strategies and identifying inherent limitations relative to how these studies represent burrow morphology. Following this, the author discusses the workflow introduced in this study, elucidating how it potentially addresses and overcomes the identified shortcomings of previous approaches regarding representation of burrow morphology.

### Historical development in numerical modelling of burrows

One of the first attempts to create a computer model of burrows was by Gingras et al.^22^. They used what they observed in sedimentary strata and techniques like resin casting to make a basic numerical model of burrows. This was then used to see how burrows might control water flows through rock layers. Their work was a big step forward, helping us move from conceptual to a quantitative understanding of how burrows control petrophysical properties of reservoirs and aquifers (Fig. 11). After Gingras et al.^22^, La Croix et al.^21^ developed a different approach to digitally model burrows (Fig. 11). They took five simple shapes, such as cylinders and lines, to represent typical burrow forms. These digital burrows were then added to a 3Dformation of dead-end pores, as highlighted

volume filled with grid cells. They adjusted the burrow positions 1000 times randomly, then, they used Monti Carlo analysis to examine how the burrow connections changed with different burrow abundance. Their study shed light on how likely burrows were to connect vertically and horizontally in relation to bioturbation intensity (Fig. 11).

In the same time of La Croix et al.^21^ paper publication, Knaust^30^ underscored the numerical models of burrows using the SBED Geomodelling software (Fig. 11). Within the SBED platform, various trace fossils, including burrows, are given distinct morphology, sizes, and variations. These burrows are then structured into an ichnofabric, accounting for multiple layers, bioturbation intensities, colonization interfaces, and overlapping patterns. Building on this workflow, Baniak et al.^10,11^ developed numerical models of burrows for subsurface hydrocarbon reservoirs. A significant jump in their work was the capability to execute fluid flow simulations on models that represented sedimentary layers with burrows, similar to the approach introduced by La Croix et al.^21^.

Eltom et al.^25^ introduced the numerical modelling of burrows using MPS techniques. As outlined above, the success of MPS lay on the construction of training image that can reproduce the morphology of geological objects. In Eltom et al.^25^, manual 3D construction was utilized to achieve detailed burrow morphology, revealing a design that presents a complex network of burrows. Within this model, burrows have been conceptualized by connecting tubes, which function as shafts and tunnels. To ensure similarity to natural *Thalassinoides*, consistent angles between these tubes were preserved, capturing the boxwork pattern with precision. Importantly, this developed model acts as a training image, enabling the reproduction of burrows in diverse abundances using MPS. The MPS results in Eltom et al.^25^ showed comparable morphology of modelled burrows with burrows found in the rock record. In following years, Eltom^13^, Eltom et al.^14^, Eltom and Goldstein^19,23^ used the MPS techniques to address many research questions using these models as digital samples of burrows. The MPS models in these studies allowed to overcome the limited availability of comprehensive samples containing the full range of variables required to describe burrow characteristics in 3D framework.

### Advantage of using MPS for modelling burrows

Numerical models of the previous studies (a prior the use of MPS techniques) offered valuable insights into how burrow characteristics such as abundance, morphology, connectivity, and size influence the petrophysical properties of bioturbated strata. However, the digital burrows in these studies were simplistic and lacked the detailed realism of natural burrows in the stratigraphic record. Using morphologies from MPS modelling could offer a more accurate representation, capturing the true appearance of burrows in real rocks.

To further refine the representation of burrows in numerical modelling of bioturbated strata, this study outlines a workflow that used burrow morphologies derived from CT scans of real rock samples. From these scans, training images are generated, which are then used to replicate burrows in varied abundance and size through MPS. Consequently, the MPS-based numerical models produced a more realistic representation of the true characteristics of burrows in stratigraphic record.

A critical aspect of the burrow, notably absent in the schematic digital representations of earlier efforts, is its tortuosity. Tortuosity stands out as an essential morphological feature to model, given its substantial influence on the heterogeneity of petrophysical properties within bioturbated strata^24^. Additionally, tortuosity plays a significant role in shaping tortuous pathways for fluid transmission and in the formation of dead-end pores, as highlighted by Gingras et al.^24^.

Moreover, MPS models use geocellular grids, making them more realistic by varying the properties within burrow models. This is different from old methods that used fixed values. The MPS models of burrows allow for better fluid flow studies within the burrows, giving researchers deeper insights into their geological roles. With these geocellular grids, investigating and measuring burrow connectivity becomes more straightforward.

### Advancement in MPS modeling

A key advancement in this study is the incorporation of CT scans for generating training images in MPS modeling. The effectiveness of MPS models is closely tied to the quality of the training images, which are essential for capturing the accurate morphology of geological objects. In earlier research, the construction of MPS models of burrows was based on training images derived from two methods: (1) using conceptual drawings to represent burrow morphology, as outlined in Eltom et al.^25^, and (2) tracing the morphology from photographs, as demonstrated in Eltom^13^. The approach presented in this paper, while building on these previous methods, takes a different and more technologically advanced route.

In this study, the author has explored the use of CT scans to create training images for burrow modeling in MPS. This method represents a notable improvement, leveraging the detailed 3D representations of burrows captured via CT imaging. This contrasts with previous approaches that relied on 2D representations, whether through conceptual sketches or photographs. By utilizing CT scans, the training images now reflect a more accurate and detailed representation of the burrows, enhancing the overall precision and authenticity of the MPS burrow models. The advancement in MPS modeling methodology in this study enriches the field of burrow modeling by offering an alternative approach that accurately captures a diverse range of burrow attributes, allowing for the precise construction of digital burrow samples.

### Guide for using MPS modelling

This study provides a clear guide for using MPS modelling in numerically model burrows. The study included a detailed process for creating MPS models of burrows, which can be seen in Fig. 1. For a better understanding, there are videos showing each step. The study also added essential data so others can replicate the methods and results in these videos. While the process has multiple steps, it’s designed to be user-friendly. By using MPS modelling, researchers can more easily study sediment layers with burrows. The research aims promote a widespread adoption of MPS modelling in ichnological studies hope this approach will lead to more implications in the field of ichnology.

### Implications

Modeling burrows using the MPS method holds significant implications for various aspects of geological research. These implications are not limited to but include: (1) the generation of digital rock samples encompassing a range of burrow attributes; (2) modeling the petrophysical properties of strata containing burrows; (3) simulating fluid flow in burrowed strata; and (4) 3D printing of hypothetical samples for physical laboratory measurements.

#### Digital rock samples

In two significant studies conducted by Eltom and Gold^26,27^, they achieved the successful generation of digital rock samples using MPS modeling. In their initial study, the authors employed manual drawing techniques to create training images for three distinct types of burrows (*Skolithos*, *Planolites*, and *Thalassinoides*) and subsequently modeled these burrows within a geocellular grid to generate 75 MPS models. Their primary objective was to investigate the variability in the connectivity of these burrows concerning their abundance, as well as to explore how these burrows could lead to the formation of different isotropic systems based on their morphology and prevalence within reservoir rocks. In their subsequent study, Eltom and Gold^26,27^ aimed to address a long-standing question related to the representative elementary volume (REV) for measuring petrophysical properties in strata containing burrows. To tackle this challenge, they created MPS models of *Thalassinoides* with varying ranges of burrow sizes and abundances. They further sampled these models using a variety of sample cross-sections, ultimately generating a total of 540 digital samples. Their initial hypothesis sought to determine whether the REV varied with the size of the burrows, their abundance, and the specific cross-sections used for sampling. As a result of their research, the authors derived an equation that provides insights into the size and abundance of *Thalassinoides*, as well as the corresponding cross-sectional samples, required to establish a connected burrow network. Conducting such studies would have been exceptionally challenging without the development and utilization of digital rock samples, highlighting the significant utility and innovation introduced by the MPS method within this field of geological research.

#### Modeling petrophysical properties

Numerous authors have undertaken the complex task of modeling petrophysical properties within reservoirs containing burrows^9–16^. Their primary objective has been to gain a comprehensive understanding of the behavior of bulk permeability within these burrowed strata, which frequently exhibit characteristics associated with dual porosity and dual permeability systems^10,11^. These systems arise due to variations in permeability between the host rock matrix and the burrows, which can take on various states, including being open, partially open, passively filled, or actively filled^9–16^. The application of MPS modeling provides two interesting approaches to address these complex scenarios.

Firstly, MPS models are constructed using geocellular grids, enabling the distribution of petrophysical properties within both the modeled burrows and the host rock matrix through various methodologies, as elaborated upon in the modeling section. Users have the flexibility to randomly distribute petrophysical properties with or without constraints by employing a rock type-based modeling approach, in software like Petrel. In this approach, each burrow and host rock matrix is considered a distinct rock type. Alternatively, another method involves assigning a single value for each of these rock types. For example, if a user aims to model the influence of *Ophiomorpha* thick walls on lateral flow, he can assign a very low permeability value (e.g., 0.01 mD) to the cells representing the *Ophiomorpha* walls within their MPS model.

Secondly, the distributed petrophysical properties within the geocellular grid can be averaged and homogenized within a single-cell model that encompasses both the burrow and host rock matrix in different proportions. Such averaging can be accomplished using various techniques, including arithmetic, geometric, and harmonic averaging methods. This approach provides insights into the potential behavior of bulk permeability within the static representation of the model.

These two approaches, offered by MPS modeling, contribute significantly to understanding the complex interactions and permeability dynamics within burrowed strata in reservoir modeling. And, again very challenging to achieve without digital models. Their utilization underscores the indispensable role of digital models in tackling such complex challenges.

#### Fluid flow simulation

Having MPS models integrated into a platform like Petrel enables easy linkage to robust fluid flow simulation software such as Eclipse. Utilizing MPS models of burrows as inputs for fluid flow simulations, researchers can explore numerous inquiries, including determining the bulk permeability of burrowed strata characterized by dual porosity or dual permeability systems. These questions can be addressed by analyzing dynamic data, such as flow rates and pressure drops obtained from well testing in the fluid flow simulations. Additional research possibilities involve investigating the influence of burrow connectivity on flow regimes by testing various scenarios with connected and disconnected burrow volumes within MPS models. Another avenue is to examine the role of the host rock matrix in the bulk permeability of reservoirs containing burrows, achieved through multiple runs of fluid flow simulations while varying porosity and permeability of the host rock matrix. The availability of realistic burrow models, facilitated by MPS, is essential for exploring such scenarios comprehensively.

#### 3D printing

MPS models can be exported in a 3D printable format, offering researchers a valuable tool to address crucial questions regarding the petrophysical properties of burrowed reservoirs. This capability facilitates a bridge between digital rock analysis and physical experimentation, effectively narrowing the gap between the two domains. For instance, scenarios involving burrow connectivity, whether omnidirectional, unidirectional, or isolated within rock matrices of varying porosity and permeability, can be modeled using MPS techniques and then transformed into 3D-printed cylinders mirroring conventional reservoir cores. Subsequent physical experiments, such as water flooding, can be conducted on these printed cylinders to elucidate the factors influencing the bulk permeability of burrowed reservoirs.

What makes this workflow particularly advantageous is the researchers' ability to continuously modify the digital samples, adjust their properties in the MPS models, and observe the resultant impacts on laboratory results. Additionally, this approach can be beneficial for situations where obtaining physical rock samples may be challenging, as is often the case with rocks containing shale or mudstone host rock matrices. With advancements in 3D printing materials that closely mimic natural rocks, this methodology can be applied to various other purposes, including investigating the effects of burrows on the mechanical properties of rocks.

### Limitations and the way forward

A clear limitation of this workflow is its reliance on commercial software, which may not be easily accessible to all researchers. Furthermore, creating training images and MPS models necessitates the use of a geocellular grid with a vast number of cells, occasionally reaching tens of millions. Managing such extensive models demands high-performance computing and can be time-consuming, sometimes taking several hours for a single MPS modelling run, depending on computer specs.

The workflow adopted utilizes a CT scan of rock containing *Thalassinoides* within the *Glossifungites* ichnofacies. Given the notable density contrast between the host rock matrix and burrow filling in this example used, the burrow network was captured with precision. However, the outcome in situations with less pronounced density contrasts remains uncertain, suggesting the need for further investigation. Additionally, this study centered on a singular burrow type. The implications of having multiple burrow types within a rock remain an open query for subsequent research. Lastly, how will MPS models adapt when using CT scans of burrows that have different morphologies from *Thalassinoides*? This too warrants exploration in future studies.

The detailed illustrations and video tutorials accompanying this manuscript are more than just supplementary materials S1; they are powerful tools for populating knowledge and techniques in the field of petroleum geology and ichnology. For researchers who do not have access to Petrel, these resources are not just informative, but they are catalysts for innovation and adaptation. They provide a clear, visual guide that can empower researchers to not only understand but also replicate and perhaps even enhance the methodology using open-source software. In doing so, it significantly broadens the impact and applicability of the workflow this study introduced, extending its reach far beyond the confines of proprietary software environments.

## Summary and conclusion

This research introduces a method to model burrows in sedimentary strata using the multipoint statistics (MPS) approach in Petrel, leveraging CT scans of rock samples. The scans create a 3D insight into burrows, leading to detailed models that reproduce burrow characteristics and their geological attributes. The entire process is documented with video tutorials and necessary datasets, aiming to promote the wider use of this MPS modelling technique and inspire future advancements. In their recent review paper on the porosity and permeability of bioturbated strata, Baniak et al.^12^ underscored the necessity for enhanced understanding of fluid flow within bioturbated reservoirs, particularly its impact on porosity and permeability. They advocated for a greater emphasis on 3D numerical models of these strata, highlighting the challenges of hands-on experiments. Within this context, MPS modelling emerges as essential tool, shedding light on the key factors shaping bioturbated reservoirs. The workflow this study introduces marks a significant leap in the numerical modelling of bioturbated reservoirs and aquifers, bridging a crucial knowledge gap and pointing the way forward in the study of bioturbated reservoirs.

### Supplementary Information

Supplementary File 1.

Supplementary Video 1.

Supplementary Video 2.

Supplementary Video 3.

Supplementary Video 4.

Supplementary Video 5.

Supplementary Video 6.

Supplementary Video 7.

Supplementary Video 8.

Supplementary Video 9.

## Data Availability

Nine videos documenting the steps to generate MPS models from CT scans and segmented CT scans file of burrows to use the training using these videos can be found as supplementary files of this study.
